# Editorial: I got flow! The flow state in music and artistic sport contexts

**DOI:** 10.3389/fpsyg.2023.1138638

**Published:** 2023-02-02

**Authors:** Michele Biasutti, Roberta Antonini Philippe

**Affiliations:** ^1^Department of Philosophy, Sociology, Education and Applied Psychology, University of Padua, Padua, Italy; ^2^Institute of Sports Science, University of Lausanne, Lausanne, Switzerland

**Keywords:** flow, music performance anxiety (MPA), optimal experiences, performance science, professional musicians

Music and artistic sports performances require the ability to master a complex integration of highly specialized perceptual, cognitive, and psychomotor skills developed over years of practice and in dynamic environments which often involve being able to deal with a large amount of pressure (Habe et al., 2019, 2021). Every instrumental or vocal performance is an event in which stakes are high and the performer has to control any single detail (Antonini Philippe et al., 2022). Several feelings are similar to most important music and sports events such as auditions and competitions. The candidates have only a few minutes to convince the jury and the “opponents” are very strong. In such situations, what role could flow have and what are the conditions to reach flow? What are the crucial concurrent variables for determining flow? How is flow regulated?

Flow is composed of cognitive, physiological, and affective factors (Biasutti, 2017; Biasutti and Habe, 2021), and is characterized by a balance between the perceived challenges or opportunities for action in the framework of existing abilities (Csikszentmihalyi, 1990). When studying flow, the self-regulation efforts that individuals use to alter their interactions with the environment and meet their goals should be considered (Sinnett et al., 2020). There is increasing evidence regarding the role of optimal experiences during music and artistic sports performances, as in the case of flow (MacDonald et al., 2006). Several other aspects should be considered in association to flow, such as anxiety. Most science-based intervention programs focusing on emotional experiences during a performance are mainly concerned with dealing with the negative symptoms and cognitive disruptions of performance anxiety (Antonini Philippe et al., 2022).

This Research Topic aims to present applied research in flow from experts within the field of performance psychology related to music and sports performance. It offers a collection of four new research studies that extend and advance our understanding of the ways that flow can influence music and sports performance. The collection draws on the work of 20 researchers from six different countries across the world representing three continents, with each article offering a representation of how flow can relate to other relevant aspects of human functioning and is specifically linked to music and sports performance. Two articles are focused on music, one on physical activity, and one merges both topics involving elite college athletes and musicians. In addition, the articles jointly illustrate a variety of contemporary research approaches including qualitative and quantitative methodologies. Evidence emerges that flow is a multidimensional construct that could be investigated from several perspectives requiring the proper methodologies.

On the music side, a quantitative approach was followed by Guyon et al. for investigating how audience and general music performance anxiety affects classical music students' flow experience. The study involved 121 university music students who performed a music piece once by themselves (private performance) and once in front of an audience (public performance). Their general music performance anxiety level was measured with an adapted version of the STAI, while the nine flow dimensions were assessed with the Flow State Scale-2 after each performance. Findings provided evidence that the effects of music performance on anxiety levels varied greatly across flow state dimensions and that musicians' flow states should be analyzed at the dimension level rather than as global scores.

The second music quantitative study is by Spahn et al. and focuses on the relationship between flow and music performance anxiety in live music performance involving 363 orchestral musicians in relation to a particular live music performance. The musicians filled out a questionnaire measuring flow experience, functional coping, perceived symptoms of music performance anxiety, and self-efficacy. The results showed that experiencing flow was on average higher among orchestral musicians compared to a sample of the general population, and differences between professional and non-professional musicians emerged. Furthermore, flow seems to have positive effects on functionally coping with music performance anxiety and self-efficacy after the performance, highlighting the negative relationship between flow and symptoms of music performance anxiety.

The physical activity study by Deng et al. emphasizes the impact of mind-wandering on flow, examining the critical role of physical activity and mindfulness involving 429 Chinese college students. The relationship between mind-wandering and flow, as well as the potential mediation effects of physical activity and mindfulness in this association, were investigated using a quantitative methodology. A cross-sectional exploratory study design was used, including multiple scales such as the Mind-Wandering Questionnaire, the International Physical Activity Questionnaire Short Form, the Mindfulness Attention and Awareness Scale, and the Short Dispositional Flow Scale. The multiple mediation model adopted to examine the relationships between mind-wandering, flow, physical activity, and mindfulness demonstrated that physical activity and mindfulness mediated the relationship between mind-wandering and flow, respectively. Evidence was provided for understanding how our minds attend to the present moment and the crucial roles of physical activity and mindfulness in the association between mind-wandering and flow.

The last article by Antonini Philippe et al. merges music and sport, presenting an exploratory investigation of elite college athletes and musicians focusing on the achievement of flow. While there are a number of studies on the characteristics of flow states and their relation to peak performance, little is known about the dynamics by which flow states emerge and develop over time. A qualitative methodology based on interviews was adopted for exploring the pre-conditions to entering flow and the development of flow over time until its termination. Three phases that the 22 athletes and musicians experienced during flow were identified: preparation to enter a flow state, entry into the flow state, and exit from the flow state. These findings provide insights into the phenomenological characteristics of the transition and maintenance of the three proposed phases and the temporal dynamics of flow.

In summary, these articles demonstrate flow is an important construct for both musicians and athletes that can have an impact on their music and sports performance. Flow can enhance the performance of musical and sports activities, whether focused on individual or collective tasks. Jointly, these studies highlight the multiplicity of ways in which music can be experienced, consider flow as an important factor for music and sports performances, and have several practical implications. For example, people that want to cultivate flow in their daily life have to decrease mind-wandering, and engagement in physical and mindfulness activities can act as preventive strategies to achieve flow. The studies reported several important achievements but, at the same time, new opportunities for further developments were considered, demonstrating that flow is a flourishing topic for research.

## Author contributions

The editorial was drafted by MB and RP. All authors listed have made a substantial, direct and intellectual contribution to the edited collection, and have approved this editorial for publication.
